# HIV and development of epithelial cell abnormalities in women with prior normal cervical cytology in Nigeria

**DOI:** 10.1186/s13027-020-00316-5

**Published:** 2020-07-29

**Authors:** Jonah Musa, Supriya D. Mehta, Chad J. Achenbach, Charlesnika T. Evans, Neil Jordan, Francis A. Magaji, Victor C. Pam, Patrick H. Daru, Olugbenga A. Silas, Atiene S. Sagay, Rose Anorlu, Yinan Zheng, Mamoudou Maiga, Isaac F. Adewole, Robert L. Murphy, Lifang Hou, Melissa A. Simon

**Affiliations:** 1 Department of Obstetrics and Gynecology, College of Medical Sciences, University of Jos, Jos, Plateau State Nigeria; 2 Department of Preventive Medicine, Division of Cancer Epidemiology and Prevention, Feinberg School of Medicine, Northwestern University, Chicago, USA; 3 Center for Global Oncology, Institute for Global Health, Feinberg School of Medicine, Northwestern University, Chicago, USA; 4 Division of Epidemiology and Biostatistics, School of Public Health, University of Illinois at Chicago, Chicago, IL USA; 5 Division of Infectious Diseases, Department of Medicine, Feinberg School of Medicine, Northwestern University, Chicago, USA; 6 Department of Preventive Medicine, Center for Health Services and Outcomes Research, Global Health, Institute for Public Health and Medicine, Feinberg School of Medicine, Northwestern University, Chicago, IL USA; 7 Center of Innovation for Complex Chronic Healthcare (CINCCH), Department of Veterans Affairs, Edward Hines Jr. VA Hospital, Hines, IL USA; 8 Department of Psychiatry & Behavioral Sciences, Feinberg School of Medicine, Northwestern University, Chicago, USA; 9 Department of Pathology, Faculty of Medical Sciences, University of Jos, Jos, Plateau State Nigeria; 10 Department of Obstetrics and Gynecology, College of Medicine, University of Lagos, Jos, Nigeria; 11 Center for Population Epigenetics, Robert H. Lurie Comprehensive Cancer Center and Department of Preventive Medicine, Northwestern University Feinberg School of Medicine, Chicago, IL 60611 USA; 12 Techniques and Technologies of Bamako, University of Sciences, Bamako, Mali; 13 Department of Obstetrics and Gynecology, College of Medicine, University of Ibadan, Ibadan, Nigeria; 14 Department of Obstetrics and Gynecology, Preventive Medicine and Medical Social Sciences, Feinberg School of Medicine, Northwestern University, Chicago, USA

**Keywords:** HIV, Normal cervical cytology, Epithelial cell abnormalities, Cervical cancer screening

## Abstract

**Background:**

HIV-associated cellular immune dysfunction has been linked to higher risk of cervical dysplasia and cancer in HIV infected women. We sought to understand the relationship between HIV and development of epithelial cell abnormalities (ECA) at follow-up in women with prior normal cervical cytology (NCC).

**Methods:**

Retrospective cohort analysis of women who received a Pap test at the Operation Stop Cervical Cancer Unit in Jos, Nigeria over a 10-year period (2006–2016). We analyzed the data of women with NCC at first Pap who had at least one follow-up cytology result for time-to-detection of ECA. We determined follow-up time in years from date of first NCC to date of first ECA report or date of last NCC follow up report with censoring at last follow-up date or December 31st, 2016 whichever came first. The primary outcome was development of any ECA as defined by the Bethesda 2001 reporting system. We identified demographic and clinical factors associated with incident ECA using multivariable Cox regression.

**Results:**

A total of 1599 women were eligible for this analysis. Overall, 3.7% (57/1556) of women reported being HIV infected. The median age at first Pap was 39 years (IQR; 33–45). The HIV infected women were younger (36.3 ± 8.1) compared to those uninfected (39.3 ± 6.6), *p* = 0.005. After an accrued follow-up time of 3809 person-years (PYs), 243 women (15%) had an ECA with an event rate of 6.38 per 100 PYs. Women ≥35 years at first Pap were more likely to have an ECA compared to those < 35 years (7.5 per 100 PYs vs 3.8 per 100 PYs, HR = 1.96; 95% CI: 1.4, 2.8). HIV status was not significantly associated with developing ECA in either unadjusted (7.4 per 100 PYs vs 6.4 per 100 PYs, HR = 1.17; 95% CI: 0.53, 2.3) or adjusted analyses (aHR = 1.78; 95% CI: 0.87, 3.65).

**Conclusion:**

Women living with HIV and on successful antiretroviral treatment may not have a differential hazard in the development of ECA during follow up after a prior normal Pap. Offering a repeat CCS to women who are 35 years or older irrespective of HIV status is likely an effective strategy in resource limited settings.

## Introduction

Although invasive cervical cancer (ICC) has a well-known natural history with treatable precancerous abnormalities detectable through screening, it is a significant public health burden in Low-and Middle-Income Countries (LMICs). A global estimate involving 185 countries reported that approximately 570,000 cases of cervical cancer and 311,000 deaths from the disease occurred in 2018 [1]. Cervical cancer was the fourth most common cancer in women and was the leading cause of cancer-related death in women in eastern, western, middle, and southern Africa [1]. It is ranked in the top three cancers affecting women younger than 45 years in 146 of 185 countries assessed [1]. Nigeria is one of these countries with a large burden of CC incidence and mortality [2].

Even with the availability and use of HPV vaccine, in LMIC’s Cervical cancer screening (CCS) is an important preventive service for reducing ICC incidence and mortality. The precancerous abnormalities detectable at screening range from minor atypical cells, low-grade epithelial cell abnormalities (ECA) to severe or high-grade ECA that could progress to ICC if not detected and treated. The reporting of these ECA detected through screening by the Papanicolou smear test (Pap test) and cytologic interpretation is guided by the 2001 Bethesda system [3].

Precancerous cervical lesions, when identified and treated early, can prevent progression to ICC. Evidence from previous studies have however, shown that development of ICC occurs at younger median ages in HIV positive women compared to women who are HIV negative [4–6]. Also, among women aged less than 35 years, being HIV positive confers a 4-fold higher risk of having ICC compared to being HIV negative [4]. The synergistic role of high-risk human Papillomavirus and HIV-associated cellular immune dysfunction has been linked to higher risk of cervical dysplasia and cancer in HIV infected women particularly in those with CD4 T cell counts below 200 cells/μL [7–12]. Since cervical cancer screening programs are limited in most resource-limited settings, and the opportunistic screening services available in Nigeria are reaching less than 9% of the population at risk [13], understanding the rate of development of incident cervical abnormalities could help guide recommendations for cervical cancer screening in such populations, particularly with respect to screening intervals for those with prior normal cervical cytology. Importantly, the rate of ECA after a prior normal cervical cytology in HIV infected women is not well-known. We sought to understand the relationship between patient-reported HIV infection and development of epithelial cell abnormalities (ECA) at follow-up test in women with normal cervical cytology (NCC) at first Pap test in Jos, Nigeria.

## Methods

### Study design and setting

We performed a retrospective cohort analysis of data on a sample of women who had received a CCS at the “Operation Stop Cervical Cancer (OSCC) Unit in, Jos, Nigeria, over a 10-year time period (2006–2016). The OSCC Unit commenced cervical cancer screening and treatment in 2006 with funding from Exxon Mobil, Texas, USA, through the African Organization for Research and Training in Cancer (AORTIC). This project offered opportunistic CCS services to women in Jos, neighboring towns, and states in northern Nigeria. The project has maintained an up-to-date electronic database and backup Paper records of all women utilizing the service. The database includes patient demographic and risk factor variables that are obtained from women at the first screening visit prior to cervical sample collection for Pap smear test. Each woman utilizing the service is given a unique medical record number, and all subsequent records including the cytopathology reports are entered into an operational database on FileMaker Pro version 8.0 [14].

### Study sample

**Fig. 1:**
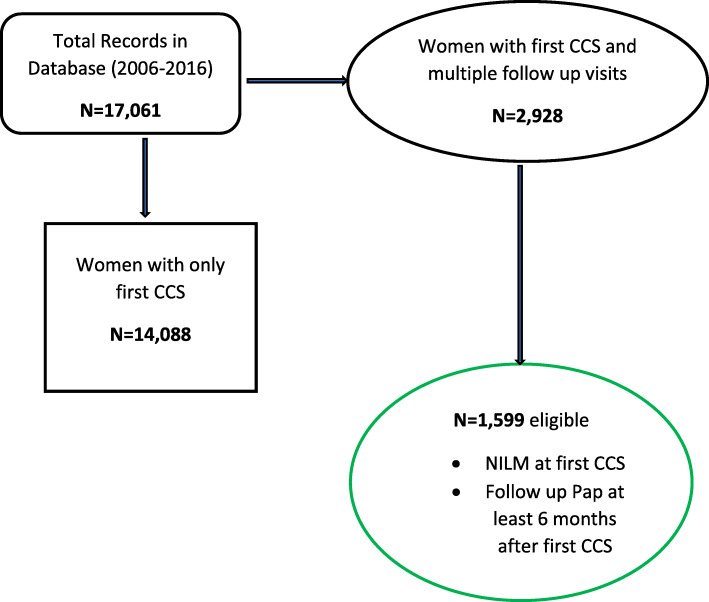
Study sample derivation from the primary sample of women with normal cervical cytology outcome at first CCS (NILM) and had at least one followed up cytology outcome (*N* = 1599)

A cohort of women who had a result of negative for intraepithelial lesion or malignancy (NILM) at the first cervical screen and had at least one follow up cytology test was included to analyze time to first detection of an epithelial cell abnormality (ECA) and the hazard associated with such abnormalities by HIV status and other patient-reported sociodemographic characteristics. The detailed description of the study sample derivation is illustrated in Fig. 1 and in the supplementary file 1 in a previous report [15]. Also, a cross-sectional analysis of the larger primary sample from which this follow up cohort was derived has been published [16].

### Statistical analysis

We analyzed the sample of women who had NILM at first Pap test and had at least one followed up cytology result for time to detection of any ECA at subsequent follow-up Pap. The observation period was from 3 January 2006 through 31 December 2016. We determined follow-up time in years from date of first NILM to the date of first ECA report or date of last NILM follow up report. The primary outcome for this analysis was development of any ECA as described by the Bethesda 2001 reporting system [3].

We performed descriptive statistics and compared means of continuous variables by outcome with the Student’s t-test, while categorical variables were compared using the Pearson Chi-Square or Fisher’s Exact test where applicable. The corresponding *p*-values were estimated, and statistical significance was set at < 0.05.

To estimate the hazard ratios for development of any ECA during follow-up, we first subcategorized the cytology report into 2 categories as follows: Negative for intraepithelial lesion or malignancy (NILM) as category 1 (referent category); any ECA report (ASCUS, LSIL, ASCUS-H, AGUS, HSIL, HSIL with suspicion for invasion) as category 2 (primary outcome). We also created dummy variables for patient-reported HIV and other socio-demographic characteristics of the study sample to estimate the effect of these covariates on the hazard of development of an ECA during the follow up period. Specifically, for patient-reported HIV, we treated HIV unknown as “missing” and categorized HIV uninfected as “referent category” and HIV infected as “primary exposure”. We also created dummy variables for other sociodemographic variables such as parity (< 3 vs ≥ = 3), age at first sex (< 20 years vs ≥ = 20 years), smoking (No vs Yes), alcohol (No vs Yes), lifetime number of sex partners (< 3 vs ≥ =3), history of vaginal infection (No vs Yes), and history of ever diagnosed with an STI (No vs Yes). We then performed bivariable and multivariable analyses using Cox regression models, with relevant Kaplan-Meier methods for selected variables. The unadjusted and adjusted hazard ratios (HRs) with their corresponding 95% CIs were estimated for patient-reported HIV and other sociodemographic variables in the sub-sample. We used the log-rank test of equality of survival function to compare differences between groups. A *p*-value of < 0.05 was considered a statistically significant difference in development of outcome event between the groups. Tests of proportional hazards assumption were based on Schoenfeld residuals; a global test p-value < 0.05 suggested violations. We used *Stata/IC Statistical Software Version 14,* College Station, TX: StataCorp USA for all statistical analyses.

## Results

During the study period, 1599 women with NILM at first Pap test had at least one follow-up Pap cytology screening test. Of the 1556 women who reported their HIV status, 3.7% (57/1556) were HIV infected. The median age at first Pap was 39 years (IQR; 33–45), and HIV infected women were significantly younger (36.3 ± 8.1) than those uninfected (39.3 ± 6.6; *p* = 0.005) at age at first Pap test. The mean follow-up time was similar for women who developed an ECA compared to those with NILM at follow up (2.3 ± 1.6 vs 2.4 ± 1.6 years, respectively: *p*-value = 0.383). The mean time between first CCS to last follow up time for HIV infected women was comparable to the HIV uninfected (2.1 ± 1.5 years vs 2.4 ± 1.6 years, respectively: p-value = 0.217). The baseline comparability of women with NILM versus women who developed an ECA at follow-up is presented in Table 1.

**Table 1:** Distribution of baseline socio-demographic characteristics by follow up cervical cytology outcome of women with normal cervical cytology category at first CCS in an opportunistic screening program in Jos, Nigeria (*N* = 1599)

Variable	Normal cytology (NILM)	Abnormal cytology (ECA)	***p***-value
**Patient-reported HIV (*N*** **= 1556)**			
Not infected	1272 (84.9)	227 (15.1)	0.894^***†***^
Infected	48 (84.2)	9 (15.8)	
**Mean follow up time in yrs (Mean ± SD)**			
**Median follow up time in yrs (IQR)**	2.4 ± 1.6	2.3 ± 1.6	0.383^a^
	2.0 (1.2–3.1)	1.9 (1.1–2.9)	
**Mean Age at first CCS (Mean ± SD)**	38.8 ± 7.9	41.9 ± 8.6	0.001^a^
**Age at first CCS (N = 1599)**			
< 35 years	422 (90.6)	44 (9.4)	0.001^***†***^
≥ 35 years	934 (82.4)	199 (17.6)	
**Age at first sex (*N*** **= 1565)**			
< 20 years	496 (83.8)	96 (16.2)	0.451^***†***^
≥ 20 years	829 (85.2)	144 (14.8)	
**Parity (*N*** **= 1488)**			
< 3	426 (90.4)	45 (9.6)	0.001^***†***^
≥ 3	830 (81.6)	187 (18.4)	
**Total life-time Sex partners (*N*** **= 1575)**			
< 3	928 (84.3)	173 (15.7)	0.364^***†***^
≥ 3	408 (86.1)	66 (13.9)	
**History of Smoking (ever) (*N*** **= 1590)**			
No	1339 (84.8)	241 (15.2)	0.536^b^
Yes	9 (90.0)	1 (10.0)	
**History of Alcohol (ever) (*N*** **= 1581)**			
No	1261 (84.5)	232 (15.5)	0.101
Yes	80 (90.9)	8 (9.1)	
**Reported history vaginal infection (*N*** **= 1560)**			
No	199 (80.6)	48 (19.4)	0.043^***†***^
Yes	1124 (85.6)	189 (14.4)	
**Ever diagnosed with STI (*N*** **= 1228)**			
No	908 (84.9)	161 (15.1)	0.844^***†***^
Yes	136 (85.5)	23 (14.5)	

^a^Student’s t-test and ^†^Pearson’s Chi-Square. ^b^Fisher’s Exact. Percent in parenthesis, *SD* standard deviation, *IQR* interquartile range. Due to incomplete responses, some of the variables may not add to the total sample of *N* = 1599)

### Unadjusted and adjusted cox-regression for development of ECA by HIV and other sociodemographic variables at follow up after normal cervical cytology at first CCS

**Fig. 2:**
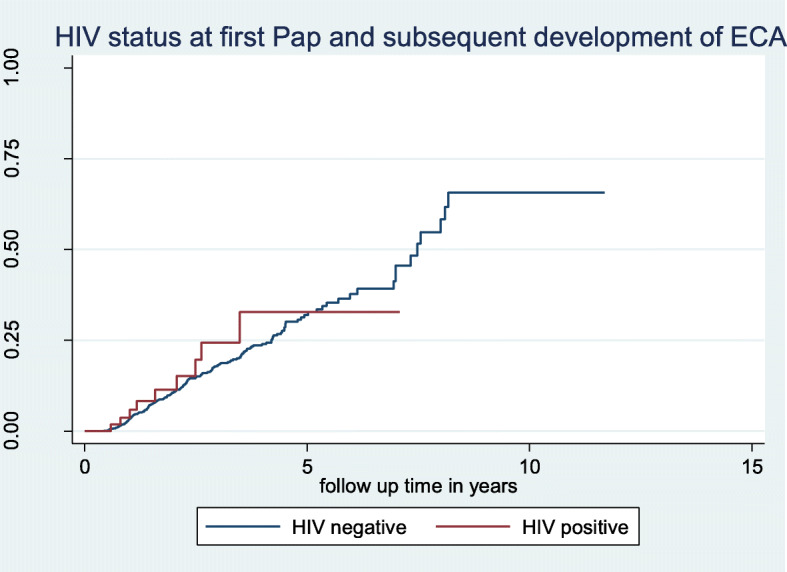
Kaplan-Meier Plot of patient-reported HIV at first cervical cancer screening with normal cytology and development of ECAs at subsequent follow up Pap cytology (log-rank test, p-value = 0.534). Proportional hazards assumption test (global test: X^2^ = 0.45, df = 1, *p* = 0.501)

**Fig. 3:**
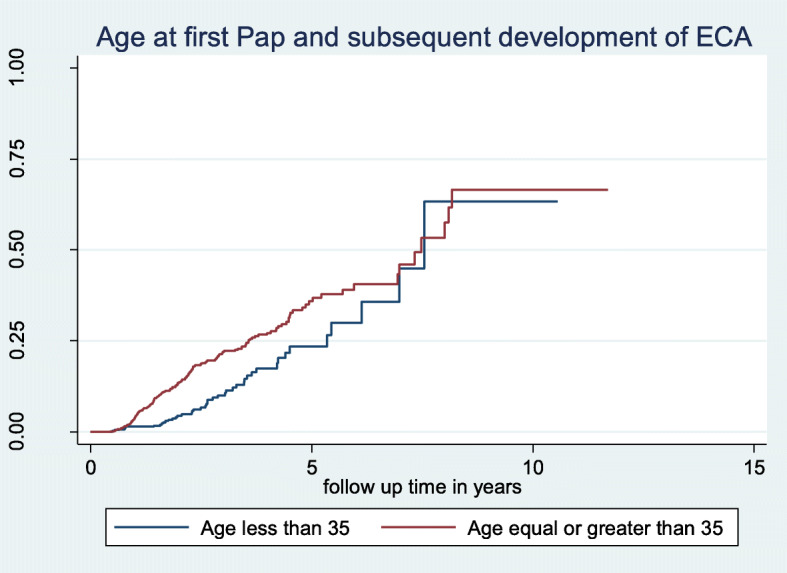
Kaplan-Meier Plot of age at first cervical cancer screening with normal cytology and development of ECAs at subsequent follow up Pap cytology (log-rank test, *p*-value = 0.001). Proportional hazards assumption test (global test: X^2^ = 8.84, df = 1, *p* = 0.0029)

After a total accrued follow-up time of 3809 person-years, 243 women (15%) had an ECA at follow up with an event rate of 6.38 per 100 person-years (PYs). Women ≥35 years old at first Pap were significantly more likely to have an ECA at follow-up Pap test compared to women younger than 35 (7.5 per 100 PYs vs 3.8 per 100 PYs, HR = 1.96; 95% CI: 1.4, 2.8). HIV infection was not significantly associated with developing an ECA during a follow up Pap test in either unadjusted (7.4 per 100 PYs vs 6.4 per 100 PYs, HR = 1.17; 95% CI: 0.53, 2.3) or adjusted analyses (aHR = 1.78; 95% CI: 0.87, 3.65). Figure 2 shows the Kaplan-Meier curve of HIV and development of an ECA at follow up with no significant difference between HIV infected and uninfected women. However, Fig. 3 shows a significant hazard for an ECA during follow-up in women age ≥ 35 years at first CCS (Log-rank *p*-value < 0.001).

**Fig. 4:**
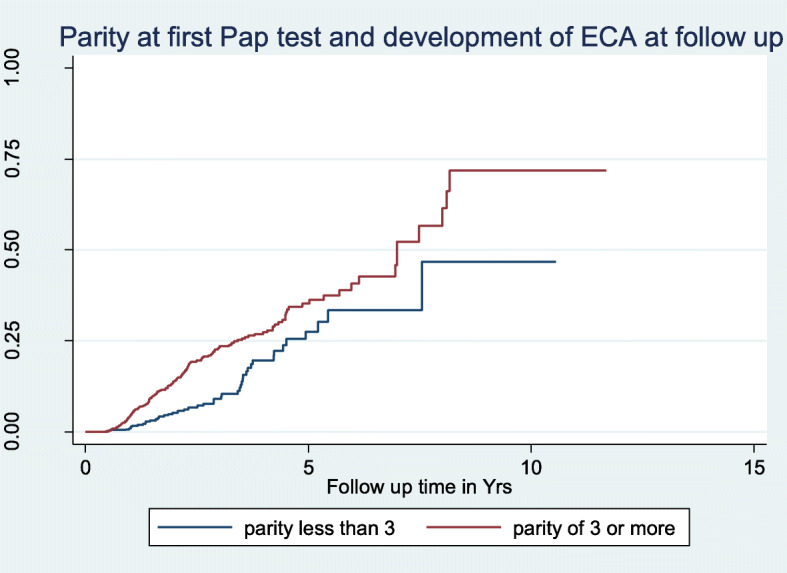
Kaplan-Meier Plot of parity at first cervical cancer screening with normal cytology and development of ECAs at subsequent follow up Pap cytology (log-rank test, p-value = 0.001). Proportional hazards assumption test (global test: X^2^ = 4.25, df = 1, *p* = 0.039)

The unadjusted and adjusted HR for development of ECA for parity of 3 or more compared to lower parity was 2.0 (95% CI: 1.45, 2.78) and 1.65 (95% CI: 1.14, 2.37) respectively. Figure 4 also showed the Kaplan Meier curve for developing an ECA with follow up Pap test by parity group (Log-rank p-value < 0.001).

The unadjusted and adjusted hazard ratios for an ECA for other sociodemographic and clinical variables of the sample are summarized in Table 2. History of vaginal infection at first Pap test was associated with a lower HR for an ECA at follow up Pap test (aHR = 0.67; 95%CI: 0.58, 0.98).

**Table 2:** Results of bivariable and multivariable Cox regression model with unadjusted and adjusted hazard ratio for development of ECA by patient-reported HIV and other sociodemographic variables at follow up after normal cervical cytology at first CCS in Jos, Nigeria (*N* = 1413 for final multivariable model)

Variable	HR (95% CI)	***p***-value	aHR (95% CI)	***P***-value
**HIV status**				
Uninfected	1.0			
Infected	1.25 (0.65, 2.41)	0.535	1.78 (0.87, 3.65)	0.116
**Age at first CCS**				
< 35 years	1.0			
≥ 35 years	1.98 (1.43, 2.75)	0.001	1.63 (1.11, 2.41)	0.013
**Parity**				
< 3	1.0			
≥ 3	2.0 (1.45, 2.78)	0.001	1.65 (1.14, 2.37)	0.008
**Age at first sex**				
> 20 years	1.0			
≤ 20 years	0.89 (0.69, 1.16)	0.391	–	–
**Total lifetime sex partners**				
< 3	1.0			
≥ 3	0.89 (0.67, 1.18)	0.426	–	–
**History of vaginal infection**				
No	1.0			
Yes	0.70 (0.51, 0.96)	0.025	0.67 (0.48, 0.93)	0.015
**Ever diagnosed with STIs**				
No	1.0			
Yes	0.97 (0.63, 1.50)	0.885	–	–
**History of Smoking**				
No	1.0			
Yes	0.50 (0.07, 3.60)	0.488	–	–
**Alcohol consumption**				
No	1.0			
Yes	0.51 (0.25, 1.02)	0.059	0.49 (0.22, 1.05)	0.067

Final multivariate Cox regression model included HIV status at first screen, age at first screening, parity, history of vaginal infection and alcohol consumption (*N* = 1413). Proportional-hazards assumption test based on Schoenfeld residual (global test: X^2^ = 18.1, df = 5, P = 0.0029)

## Discussion

This retrospective cohort analysis has contributed to the understanding of the clinical and epidemiologic characteristics that are associated with incident ECA in women with a prior normal cervical cytology attending an opportunistic cervical cancer screening in Jos, Nigeria. It also provides us with evidence that though HIV infection is known to be associated with higher prevalence of precancerous lesions of the cervix and to also accelerate progression to invasive cancer stages, other clinical and sociodemographic factors are important contributors to these associations. Our data suggest that sociodemographic variables such as late age at first screening and multiparity are significant factors for development of ECAs during follow-up in women who had prior normal cervical cytology.

Our principal finding showed that self-reported HIV status at first CCS was not significantly associated with the development of ECA at follow up Pap test (aHR = 1.78; 95% CI: 0.87, 3.65). Studies on the hazard of any squamous intraepithelial lesions in HIV positive women compared to HIV negative have shown no significant difference particularly if the CD4+ count level is ≥500 cell/μL [17]. A similar study that followed women with a prior normal Pap cytology over a 24 month period in South Africa found that women with incident HIV infection during the follow-up period were 6 times more likely to have abnormal cervical cytology [18]. The higher risk of abnormal cervical cytology in women with incident HIV infection could be explained by the high viral load with depletion in CD4+ count following incident HIV infection prior to antiretroviral therapy [19]. Although, our analysis did not adjust for the biological variables like CD4+ cell count or HIV viral load, we know that most of the HIV infected women in our sample have been on successful antiretroviral therapy supported by the Presidential Emergency Plan For AIDS Relieve (PEPFAR) adult HIV treatment program in Jos [20–23]. This assumption is supported by follow-up data in a US population that did not find a significant difference in incident cervical dysplasia and cancer in HIV population on successful HAART [24, 25]. Also, recent data from the United Nations on AIDS (UNAIDS) showed that over 80% of HIV infected adults on antiretroviral treatment in Nigeria had HIV viral loads that were suppressed [26]. These findings therefore suggest that cervical cancer screening policy should be similar irrespective of HIV status particularly where the HIV infected women are on successful antiretroviral treatment in the population. It has also been shown that whereas annual Pap test is recommended for HIV positive women, recommending longer screening intervals in HIV infected women with serial negative Pap tests and who are receiving antiretroviral treatment with low viral loads may be appropriate [27].

We also found that age at first CCS ≥ 35 years and multiparity were significantly associated with higher hazard for development of an ECA at a subsequent follow-up Pap test after prior normal cytology outcome. Our data showed that on the average women who were ≥ 35 years at first Pap test with normal cytology were 1.63 times more likely to develop an ECA at follow-up Pap test compared to women who were younger. This hazard was maintained for at least 5 years during follow up before the difference started to converge (Fig. 3). The possible explanation for the observed convergence could be related to the documented higher risk of development of ECA with advancing age with longer follow up Pap test [16]. Also, our analysis focused on the age at first CCS rather than adjusting for change in age at the time of outcome assessment. In future research, data on the age at outcome assessment should be collected and modelled as a time-varying covariate to have a better understanding of the effect of age on development of ECA.

Similarly, women with higher parity ≥3 at first Pap test with normal cytology were 1.65 times more likely to develop an ECA at follow-up Pap test compared to women with a lower parity. We have previously documented in the same screening population in Jos that cervical cancer screening at age 35 or more, and in women with high parity was associated with higher odd for an underlying abnormal cervical cytology outcome irrespective of HIV status [16]. The findings of the effect of multiparity on the development of an ECA on subsequent follow up Pap test is corroborated by reports on the cofactors in cervical pre-cancer and progression to invasive cervical cancer that women of parity 3 or more were significantly more likely to have pre-cancer compared to nulliparous women [28]. In resource limited countries like Nigeria, where organized CCS is lacking, and coverage is less than 9% of the population at risk [13] the policy implication of these findings may include recommending a single opportunity for cervical cancer screening for multiparous women at 35 years or older. This policy could be more effective in focusing cervical cancer screening resources and efforts in detecting precancer in women with known high-risk epidemiologic characteristics. These data also imply that recommending a repeat Pap test after a normal cervical cytology in women who are younger than 35 years with low parity may be less efficient strategy where resources are limited. Overall, this policy recommendation falls within the WHO concept of more women in the population having a minimum of 1 adequate Pap smear per lifetime in women older than 35 years of age [29].

Our data revealed an interesting but controversial finding that history of vaginal infection at first Pap test was associated with a 33% lower hazard for an ECA at follow up Pap test. This finding contradicts the well-known evidence that Bacterial vaginosis and sexually transmissible infections are associated with persistent high-risk human Papillomavirus and precancer [30, 31]. It is possible that reporting bias may have affected the analysis for vaginal infection given that over 80% of the women in the sample reported having vaginal infection. This is possible since, anecdotally, women in our population often perceive vaginal discharge as “toilet” infection. Since these infections were neither confirmed microbiologically nor classified as Bacterial vaginosis, Trichomonas, or Candida, it is likely that reporting bias was not controlled leading to misclassification. However, since women with vaginal infection are more likely to have had cervical cancer screening at an early age [16] the odds for an abnormal cervical cytology could be lower at first Pap test and at follow up Pap particularly if the interval was shorter.

The main strength of this analysis is the relatively large sample size, with precise estimates of the association of HIV status and other covariates on incident epithelial cell abnormality in women with a prior normal cervical cytology. This data provides baseline evidence that could guide health policy decision makers in making cervical cancer screening recommendations for both HIV and non-HIV infected women population in Nigeria and similar settings in Africa. We, however, recognize the limitations of self-reported data in this analysis. It is possible that recall bias may have led to misclassification of some variables thereby affecting the validity of our statistical estimates. We also recognize that our statistical estimates are potentially biased by some missing data, and our findings are based on reports from a population of women who were able to overcome barriers to access and received a cervical cancer screening. Therefore, our findings may not be representative of the larger population of women, particularly those in rural and suburban communities in Nigeria. We also acknowledge the limitation of aggregating different categories of ECAs as a single outcome rather than stratifying as low-grade and high-grade cervical intraepithelial lesions. This subgroup analyses will require a larger and more balanced sample size in both the HIV negative and HIV positive groups, with a longer follow-up time to have more events in both groups. A future population-based study of a nationally representative sample of Nigerian women in urban and rural settings with a longer follow-up time could provide more generalizable evidence for comparing the effect of HIV on the event rate of epithelial cell abnormalities and invasive cervical cancer in Nigeria.

## Conclusion

In conclusion, though HIV infection is known to drive the development of ECA and progression to invasive cervical cancer, our data support current evidence that HIV infected women on successful antiretroviral treatment with viral suppression may not have a differential hazard in the development of ECA during follow up Pap after having an initial normal Pap test result. Secondly, our data suggests that in resource poor countries like Nigeria, where organized CCS is lacking, recommending a single opportunity for cervical cancer screening for multiparous women at 35 years or older could be an efficient screening strategy to maximize use of limited resources. These data also imply that recommending a repeat Pap test after a normal cervical cytology in women who are younger than 35 years with low parity may not be an efficient strategy where resources are limited.

## Data Availability

All the relevant data for this analysis have been presented in the body of this manuscript and the associated tables and figures. The original data sources and the dataset used in this analysis is available upon reasonable request to the corresponding author.
